# Inequitable Access to Health Care by the Poor in Community-Based Health Insurance Programs: A Review of Studies From Low- and Middle-Income Countries

**DOI:** 10.9745/GHSP-D-16-00286

**Published:** 2017-06-27

**Authors:** Chukwuemeka A Umeh, Frank G Feeley

**Affiliations:** a Department of Global Health, Boston University School of Public Health, Boston, MA, USA.

## Abstract

**Background:**

Out-of-pocket payments for health care services lead to decreased use of health services and catastrophic health expenditures. To reduce out-of-pocket payments and improve access to health care services, some countries have introduced community-based health insurance (CBHI) schemes, especially for those in rural communities or who work in the informal sector. However, there has been little focus on equity in access to health care services in CBHI schemes.

**Methods:**

We searched PubMed, Web of Science, African Journals OnLine, and Africa-Wide Information for studies published in English between 2000 and August 2014 that examined the effect of socioeconomic status on willingness to join and pay for CBHI, actual enrollment, use of health care services, and drop-out from CBHI. Our search yielded 755 articles. After excluding duplicates and articles that did not meet our inclusion criteria (conducted in low- and middle-income countries and involved analysis based on socioeconomic status), 49 articles remained that were included in this review. Data were extracted by one author, and the second author reviewed the extracted data. Disagreements were mutually resolved between the 2 authors. The findings of the studies were analyzed to identify their similarities and differences and to identify any methodological differences that could account for contradictory findings.

**Results:**

Generally, the rich were more willing to pay for CBHI than the poor and actual enrollment in CBHI was directly associated with socioeconomic status. Enrollment in CBHI was price-elastic—as premiums decreased, enrollment increased. There were mixed results on the effect of socioeconomic status on use of health care services among those enrolled in CBHI. We found a high drop-out rate from CBHI schemes that was not related to socioeconomic status, although the most common reason for dropping out of CBHI was lack of money to pay the premium.

**Conclusion:**

The effectiveness of CBHI schemes in achieving universal health coverage in low- and middle-income countries is questionable. A flexible payment plan where the poor can pay in installments, subsidized premiums for the poor, and removal of co-pays are measures that can increase enrollment and use of CBHI by the poor.

## Abstract (precis)

The poor lack equitable access to health care in community-based health insurance schemes. Flexible installment payment plans, subsidized premiums, and elimination of co-pays can increase enrollment and use of health services by the poor.

## INTRODUCTION

The World Bank defines the poor as individuals or families who do not have the resources or abilities to meet their daily needs.^1^ Poverty is usually associated with poor health outcomes, with the poorest of the poor having the worst health outcomes.^2^^,^^3^ Furthermore, the poor are most disadvantaged by out-of-pocket expenditures, and they are the ones who are most likely to be ill and less able to afford to pay for health care.^4^^,^^5^ To ensure that the poor have access to health care when they need it and that they are protected from catastrophic health expenses, health systems need to be financed by either tax or prepayment schemes.^1^^,^^3^ The prepayment scheme should lead to a large risk pool and enough money in the health system to cross-subsidize the sick and the poor. While health insurance schemes are the norm in high-income countries, the story is different in low-income countries.^1^^,^^3^

In light of this, there is a push to encourage countries to provide access to basic health care for all their citizens through prepayment or tax schemes. A general taxation scheme would be most desirable, but this is not feasible in several low- and middle-income countries because people are poor and many work in the informal sector, making revenue collection difficult.^6^ This has led some poor countries to choose community health insurance schemes as an alternative way of providing access to basic health care for those in rural communities and the informal sector.^7^^,^^8^

Some poor countries have chosen community health insurance schemes as a way of providing access to basic health care for those in rural communities and the informal sector.

Community-based health insurance (CBHI) schemes refer to voluntary, nonprofit health insurance schemes organized and managed at the community level. While CBHI schemes vary in design and implementation, all are based on the principle of risk pooling and involve regular payments of a small premium in exchange for reducing direct payments at the point of service.^9^ This is important because direct payment at the point of service has been shown to delay or deter the use of health services.^10^^,^^11^

CBHI schemes share 3 common characteristics: they include (1) not-for-profit prepayment plans, (2) community control, and (3) voluntary membership.^12^ CBHI schemes have been shown to improve use of health services among children and pregnant women^13^^,^^14^ and to reduce catastrophic health expenditure.^15^^,^^16^ Catastrophic health expenditure results in families cutting down on other necessities such as food, clothing, and children's education, and its impact is greatest for the poorest families.^4^ In a cross-country analysis, Xu and colleagues noted that catastrophic payments would be reduced if health systems relied less on out-of-pocket payments.^17^

Community-based health insurance schemes include not-for-profit prepayment plans, community control, and voluntary membership.

The focus of this article is on demand for CBHI by the poor. Demand for health insurance is influenced by the benefit consumers expect to derive from health insurance, by the amount they are expected to pay as premium, and by their income. Additionally, demand for health insurance is influenced by consumers' probability of getting sick (with the elderly and chronically ill more likely to sign up for insurance) and their aversion to risk. Risk-averse consumers are willing to pay higher premiums to avoid the risk of a greater loss.^18^ Thus, consumers will purchase CBHI if the expected benefits exceed the benefits of out-of-pocket payment.^18^^,^^19^

Demand for health insurance is negatively influenced by other factors such as inadequate knowledge or awareness of the existence of a health insurance scheme and how to enroll in the scheme,^20^^,^^21^ actual or perceived poor quality of health services,^22^^,^^23^ inconvenient enrollment process,^22^ inadequate benefit package,^22^^,^^24^^,^^25^ long distances to health facilities,^22^ negative provider attitude,^22^ lack of trust in CBHI officials,^21^^,^^22^^,^^26^ lack of provider choice,^24^ low education status,^27^ positive perception of the adequacy of traditional care,^27^ and a low proportion of children living within a household.^27^

### Objectives of the Present Study

Although CBHI has been shown to be helpful in increasing the use of health services and reducing catastrophic health expenditure,^13^^–^^16^ there is need to better understand how CBHI affects the poor who ordinarily should benefit more from the health insurance scheme. This review seeks to explore the:

- Effect of socioeconomic status on willingness to join and pay for CBHI
- Effect of socioeconomic status on actual enrollment in CBHI
- Effect of socioeconomic status on use of CBHI by enrollees
- Effect of socioeconomic status on drop-out rate from CBHI schemes

Although there have been previous reviews of health insurance schemes in low- and middle-income countries, to the best of our knowledge this is the first review that is focused on the effect of socioeconomic status on willingness to enroll, actual enrollment, and use of CBHI in low- and middle-income countries.

This review focuses on the effect of socioeconomic status on willingness to enroll, actual enrollment, and use of community-based health insurance in low- and middle-income countries.

## METHODS

The authors searched PubMed, Web of Science, African Journals OnLine, and Africa-Wide Information (the latter incorporating South African Studies, African Studies, and African HealthLine) for studies on willingness to enroll in CBHI, enrollment in CBHI, use of services by CBHI enrollees, and drop-out from CBHI. We also searched a collection of articles on health financing for the poor published by the World Bank, *Health Financing for Poor People: Resource Mobilization and Risk Sharing*,^28^ for relevant articles/chapters. The search terms we used included: (1) willingness to pay AND community health insurance; (2) community health insurance AND low and middle income countries; (3) community health insurance AND utilization of health services; (4) community health insurance AND drop out; (5) community health insurance AND premium AND subsidy; and (6) community health insurance AND enrollment.

We conducted the literature search in August 2014 and restricted our search to studies published between 2000 and August 2014 to obtain current information on CBHI. In addition, we restricted our search to articles written in English. Furthermore, we searched the reference list of identified articles for additional resources.

After we completed the literature search, we reviewed all the articles based on our predetermined inclusion criteria. The inclusion criteria were that the study must have been conducted in low- and middle-income countries and involved analysis based on socioeconomic status. Varied measurement of socioeconomic status was accepted, including self-reported income, assessment of assets, self-reported expenditures, and community wealth ranking (whereby community members categorize families into different wealth categories). There was also no restriction on the type of study that was included in the review. The titles and abstracts of articles were first reviewed based on our inclusion criteria. The full text of selected articles that met the inclusion criteria were then reviewed in full.

We developed a data extraction sheet, and one author extracted the data from the included studies while the second author reviewed the extracted data. Disagreements were mutually resolved between the 2 authors. Data were extracted from the included studies on: (1) characteristics of the study (including country where study was conducted, date of data collection, sample size, setting of study [urban or rural], and study design); and (2) the findings of the study. The findings of the studies in the different subsections were analyzed to identify their similarities and differences and to identify any methodological differences that could have accounted for contradictory findings.

## RESULTS

### Study Selection

A total of 49 articles were included in the review. Our initial search of the relevant databases and other sources yielded 755 articles. After removing duplicates, 722 articles remained. Of these, 645 articles were excluded after screening the titles and abstracts because they did not meet our inclusion criteria. The full text of the remaining 77 articles were evaluated in more detail, of which 34 were excluded because they did not include analysis based on socioeconomic status. The remaining 43 articles were included in the review, as were an additional 6 articles that were identified in the reference lists of the 43 included articles (Figure).

49 articles were included in this review.

**FIGURE:**
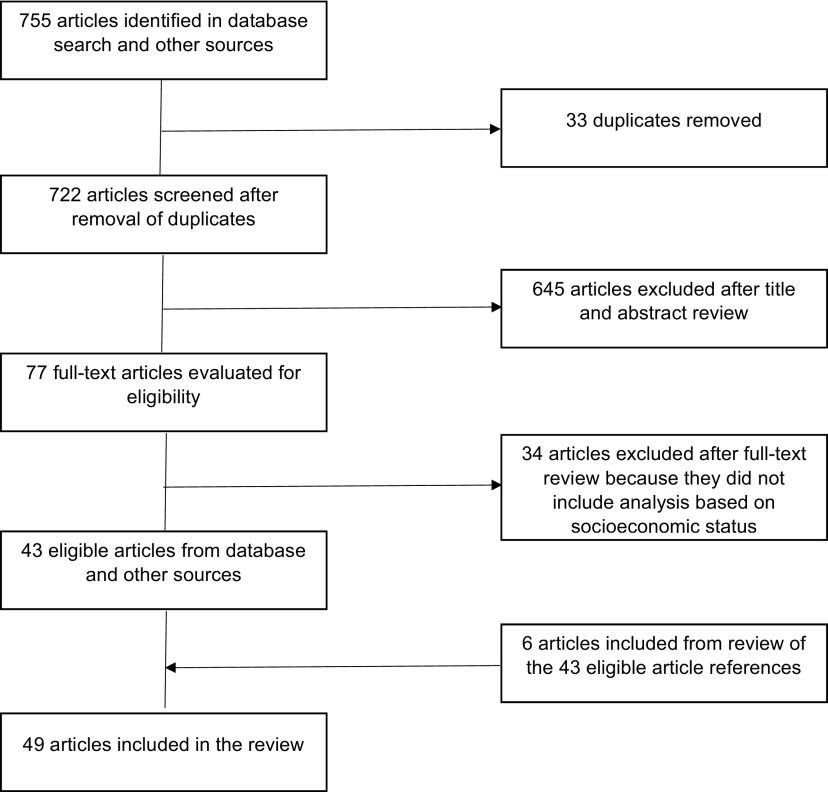
Summary of Search Results

The studies that were finally selected for review used a range of study designs, including pre- and post-test with control, pre- and post-test without control, post-test with control, post-test without control, and cross-sectional community-based pre-intervention surveys. The selected studies were conducted in Africa (in countries such as Burkina Faso, Nigeria, and Senegal); Asia (in countries such as China, India, and the Philippines); and South America (Ecuador).

### Socioeconomic Status and Willingness to Join or Pay for CBHI

There were mixed results on the effect of socioeconomic status on willingness to join a CBHI program (Table 1). Several studies, including those conducted in Ethiopia,^29^^–^^31^ China,^32^ India,^33^ and Cameroon,^34^ found that socioeconomic status was positively associated with willingness to pay, with the rich more willing to pay for CBHI than the poor. Study participants consisted of those who did not presently have health insurance. A 2007 cross-sectional survey in southwest Ethiopia found that households in the highest quintile were 2.7 times more willing to join a CBHI program than families in the lowest quintile.^31^ A more recent survey in southwest Ethiopia in 2013 showed that households in the highest wealth quintile were more than 4 times more willing to join the CBHI compared with households in the second wealth quintile.^29^ Similarly, Asfaw et al. found that a 1% increase in income in rural Ethiopia led to an 8.4% increase in the probability of willingness to pay for health insurance.^30^ In India, households in the highest wealth quintile were 2.1 times more willing to pay for CBHI compared with those in the lowest quintile.^33^ Similarly, in China, Zhanga et al. found that willingness to join a CBHI program increased by 0.83% to 1.54% when income increased by 100 Yuan in a year. The farmers who owned luxury assets were 1.37 to 1.66 times more likely to join a CBHI program than those who did not own such assets.^32^ That wealthier households are more willing to join or pay for CBHI is expected and not surprising.

**TABLE 1.** Summary of Studies on Willingness to Join or Pay for Community-Based Health Insurance

Study	Country	Date of Data Collection	Sample Size^a^	Urban/Rural	Study Design	WTJ/P
**Positive Association Between Socioeconomic Status and WTJ/P**						
Haile M et al. (2014)^29^	Ethiopia	2013	845	Rural	Cross-sectional community based survey	WTJ was 4.2 times higher in richest vs. 2nd poorest quintile (95% CI: 1.6, 10.9)
Asfaw A et al. (2004)^30^	Ethiopia	2000, 2001	550	Rural	Cross-sectional community based survey	1% increase in income increased the WTP by 8.4%
Ololo S et al. (2009)^31^	Ethiopia	2007	803	Urban	Cross-sectional community based survey	WTJ was 2.7 times higher in richest vs. poorest quintile. (95% CI: 2.1, 6.7)
Zhang L et al. (2006)^32^	China	2002	2,830	Rural	Cross-sectional household survey	WTJ was 1.37–1.66 times higher among farmers who owned luxury assets vs. those who did not
Ghosh S et al. (2011)^33^	India	NS	1,502	Urban	Cross-sectional household survey	WTP was 2.1 times (*P*=.07) higher in richest vs. poorest quintile
Dong H et al. (2005)^40^	Burkina Faso	2001	2,414	NS	Cross-sectional household survey	WTP was 1.7 times higher in richest vs. poorest (*P*<.01)
Onwujekwe O et al.^42^	Nigeria	NS	450	Both	Cross-sectional household survey	WTP was 1.8 times higher in richest vs. poorest (*P*=.001)
Onwujekwe O et al. (2010)^43^	Nigeria	NS	3,070	Both	Cross-sectional household survey	WTP was 1.7 times higher in richest vs. poorest quartile
Babatunde OA et al. (2012)^45^	Nigeria	NS	360	Rural	Cross-sectional household survey	WTP was 2 times higher in richest vs. poorest quartile
Gustafsson-Wright et al. (2009)^46^	Namibia	2008	1,750	NS	Cross-sectional household survey	WTP was 2.6 times higher in richest vs. poorest quintile; richest willing to pay 1.2% of income while poorest willing to pay 11.4% of income
Dror DM et al. (2007)^47^	India	NS	3,024	Both	Cross-sectional household survey	WTP was 2 times higher in richest vs. poorest
Binnendijk B et al. (2013)^48^	India	2008–2010	7,874	Rural	Cross-sectional household survey	Richest willing to pay more than poorest but poorest willing to pay higher proportion of total income
Shafie AA et al. (2013)^49^	Malaysia	2009	472	NS	Cross-sectional household survey	WTP was 2 times higher in richest vs. poorest quintile
Parmar D et al. (2014)^51^	Burkina Faso	2004–2008	6,827	Both	Cross-sectional household survey	WTJ was 0.27 lower in poor vs. rich (*P*=.001)
**Negative Association Between Socioeconomic Status and WTJ/P**						
Oriakhi HO et al. (2012)^36^	Nigeria	NS	360	Rural	Cross-sectional household survey	WTJ was 0.66 times lower in high- vs. low-income groups
**Mixed Results or No Association**						
Bukola A (2013)^35^	Nigeria	NS	900	Both	Cross-sectional household survey	53% decrease in WTP with 1 unit increase in income quintile in rural areas; conversely, 77% increase in WTP with 1 unit increase in income quintile in urban areas
Eckhardt M et al. (2011)^36^	Ecuador	2006	153	Rural	Cross-sectional household survey	No difference in WTJ by income groups (*P*=.23)

Abbreviations: CI, confidence interval; NS, not stated in article; WTJ, willingness to join; WTP, willingness to pay.

^a^ Sample size is the number of households.

Several studies found that socioeconomic status was positively associated with willingness to pay for community-based health insurance.

However, studies in Nigeria^35^^,^^36^ showed that the rich in rural areas were significantly less willing to pay for CBHI than the poor. A cross-sectional survey in the south-south region of Nigeria showed that respondents with lower income were 1.4 times more willing to join a CBHI program than those with higher income.^36^ Another study in southwest Nigeria showed that income was negatively associated with willingness to pay for CBHI in rural areas while it was positively associated in urban areas where more services were available and costs were higher. A unit increase in income quintile decreased willingness to pay by 53% in rural areas while it increased it by 77% in urban areas. A possible explanation for the results in these 2 Nigerian studies is that there might be low-quality services in rural health centers and so the rich prefer to travel to urban areas where they will get better services. Another possible explanation is that health services in rural areas are usually less expensive and of lower quality than in urban areas, and so the rural rich might feel they are able to pay their health bills out of pocket anytime the need arises.^35^

A study in rural Ecuador did not find any association between wealth and willingness to join a CBHI program, although those who were less educated were more willing to join.^37^ However, in Ecuador at the time of the study, one health insurance scheme covered most workers in the formal sector while another insurance scheme covered the rural population. The scheme for the rural population was noted for low-quality services, although neither of the schemes was operational in the rural village where the study was carried out. The low-quality services associated with the insurance scheme in rural areas might have explained the reason why the more educated in the rural areas were less willing to join the CBHI scheme.

The amount that individuals and families are willing to pay as premium appears to be directly related to the socioeconomic status of the individuals/families. Studies from Burkina Faso,^38^^–^^41^ Nigeria,^42^^–^^45^ and Namibia^46^ showed that the rich were willing to pay a higher premium than the poor. However, the poor were willing to pay a higher percentage of their income as premium.^46^^–^^48^

The premium amount that individuals and families were willing to pay appears to be directly related to their socioeconomic status.

In Nigeria,^42^^,^^43^^,^^45^ Burkina Faso,^38^^–^^41^ and Malaysia^49^ families/individuals in the highest wealth quintile were willing to pay a premium 1.6 to 2 times higher than those in the lowest quintile, while in Namibia those in the richest quintile were willing to pay a premium 2.6 times as much as those in the poorest quintile.^46^ The higher disparity between what the rich and poor in Namibia are willing to pay is consistent with the wealth disparity in Namibia, which has about the highest Gini coefficient in the world.^50^

Conversely, the poor are willing to pay a higher proportion of their income as premium. In India, those in the lowest income quintile were willing to pay 1.8% of their income as premium compared with 0.84% for those in the highest income quintile.^47^ However, in Namibia those in the richest quintile were only willing to pay 1.2% of their income as premium, while those in the poorest quintile were willing to pay about 11% of their income on premium.^46^ In general, willingness to pay is higher among the rich, but the poor are willing to pay a higher percentage of their income.

#### Willingness to Enroll in CBHI and Preferred Method of Premium Payment

In-depth interviews conducted in India^69^ and Burkina Faso^70^ showed that the poor prefer monthly premium payments to yearly payments because they do not have the money to pay the yearly premium at one time. Another study in Ethiopia showed that, in general, 95% of those who were willing to join the CBHI preferred to pay a monthly premium instead of yearly premium.^31^

Furthermore, studies in Nigeria showed that the rural poor would be more willing to enroll in a CBHI scheme if they were given the option of paying their premiums using commodities. In a study in southwest Nigeria, respondents in rural areas were willing to pay 2.6 times more if they were to pay in-kind, rather than with cash. Conversely, respondents in urban areas were willing to pay 0.8 times less if they were to pay in-kind instead of cash. It is important to note that 61% of the rural respondents were in the lowest 2 wealth quintiles.^71^ A similar study in southeast Nigeria showed that rural households were willing to pay a premium that was 2 times as high if they were to pay with commodities instead of cash.^44^^,^^72^^,^^73^ The preference to pay monthly premiums instead of yearly premiums or to pay premiums using commodities might be due to the inability of poor rural families to save enough money to pay the yearly premium at once.

#### Willingness to Enroll in CBHI and to Cross-Subsidize the Poor

Studies in Nigeria and Tanzania showed that the rich are willing to pay a higher premium to cross-subsidize the poor. In a study in southeast Nigeria, 53% of the respondents were willing to contribute money to cross-subsidize the poor, with 75% of those in the richest quartile willing to cross-subsidize the poor.^42^ A similar survey in Tanzania showed that 46% of rural dwellers and 41% of urban dwellers were willing to cross-subsidize the poor. However, urban households were willing to pay a higher amount to cross-subsidize the poor compared with rural households, presumably because the urban wealthy have more income than the rural wealthy.^68^

Some studies have shown that the rich are willing to pay a higher premium to cross-subsidize the poor.

In summary, studies on socioeconomic status and willingness to join a CBHI scheme suggest that in the absence of factors that might negatively affect demand for insurance (such as actual or perceived poor quality of health services),^22^^,^^23^ willingness to join a CBHI scheme is directly related to family income. Higher family income is also associated with greater willingness to pay a higher premium to cross-subsidize the poor. Additionally, willingness to join a CBHI scheme increases when families are offered flexible premium payment options such as monthly premium payments and payment using commodities.

### Socioeconomic Status and Actual Enrollment Into CBHI

In most studies, actual enrollment in CBHI was directly associated with socioeconomic status (Table 2), meaning that although a lot of the poor are willing to join CBHI, most of them do not because they cannot afford to pay the premium. Studies conducted in Burkina Faso,^27^^,^^51^ Senegal,^8^^,^^52^ the Philippines,^53^ Uganda,^54^^,^^55^ and Mali^56^ support this finding. In Burkina Faso, the poor were 73% less likely to enroll in CBHI than the rich.^51^ Another study in Burkina Faso showed that the rich were more likely to be insured than the poor, with the median household expenditure (a proxy for household wealth) 2.6 times higher among those who were insured than among the uninsured.^27^ These findings are not surprising because the rich have more money at their disposal to pay the premium than the poor.

**TABLE 2.** Summary of Studies on Enrollment in Community-Based Health Insurance

Study	Country	Date of Data Collection	Sample Size	Urban/Rural	Study Design	Enrollment
**Poor Less Likely Than the Rich to Enroll**						
Parmar D et al. (2014)^51^	Burkina Faso	2004–2008	990 households	Both	Pre and post without control (repeated measures)	The poor were less likely to either enroll or use CBHI
Jutting JP (2004)^52^	Senegal	2000	346 households	Rural	Post without control	Higher-income group significantly more likely to enroll in health insurance
Dror DM et al. (2005)^53^	Philippines	2002	1,953 households		Post with control	The poor were more uninsured than the rich
Basaza R et al. (2007)^54^	Uganda	2004–2005	63 individuals	Rural	Case study with key informant interviews	Inability to pay premium most common reason (80%) for non-enrollment
Basaza R et al. (2008)^55^	Uganda	2005–2006	185 individuals	Rural	Qualitative—focus group discussions and in-depth interviews	Inability to pay premium most common reason for non-enrollment
Franco LM et al. (2008)^56^	Mali	2004	2,280 households	Both	Post with control	Enrollment was significantly higher in the rich wealth quintile than other quintiles; insured were more likely to use health services
Saksena P et al. (2011)^58^	Rwanda	2005–2006	6,800 households	Both	Post with control	Poorer households were less likely to be insured
De Allegri M et al. (2013)^28^	Burkina Faso	2004	547 households	Both	Post with control	Enrollees in insurance scheme were more likely to be wealthier than non-enrollees
Jütting JP (2004)^9^	Senegal	2000	346 households	Rural	Post with control	The poor were less likely to enroll in CBHI
**No Association Between Socioeconomic Status and Enrollment**						
Schneider P et al. (2004)^57^	Rwanda	2000	2,518 households	Rural	Post with control	No relationship between socioeconomic status and enrollment in health insurance or use of it by enrollees
**Premium Subsidy Increased Enrollment**						
Oberländer L et al. (2014)^59^	Burkina Faso	2008–2009	25,494 individuals	Both	Regression discontinuity	Probability of enrollment increased by 30 percentage points with eligibility for premium subsidy
Parmar D et al. (2012)^60^	Burkina Faso	2004–2007	990 households	Both	Pre and post without control (repeated measures)	With onset of subsidy, percentage of the insured who were poor increased from 3.4% in 2006 to 26.0% in 2007
Souares A et al. (2010)^61^	Burkina Faso	2006–2007	7,122 households	Both	Pre and post without control	With the onset of subsidy in 2007, the proportion of the poor enrolled in CBHI increased from 1.1% in 2006 to 11.1% in 2007
Zhang L et al. (2008)^74^	China	2004–2006	1,169 households	Rural	Post without control (repeated measures)	Low-income group was less likely to enroll in the subsidized CBHI than the middle- and high-income groups
Wagstaff A et al. (2007)^75^	China	2003, 2005	8,476 households	Rural	Pre and post with control (propensity score matching)	Subsidized insurance improved use of services in the poorest 10% of the population

Abbreviation: CBHI, community-based health insurance.

In most studies, actual enrollment in community-based health insurance was directly associated with socioeconomic status.

However, a study in Rwanda in 2000 did not show any relationship between socioeconomic status and enrollment into the CBHI scheme.^57^ The explanation given for this was that the CBHI scheme in Rwanda allowed households to pay the premium in installments and households were enrolled as full members once they completed paying the premium. This encouraged the poor to enroll. In addition, churches and community members helped to pay enrollment fees for the poor, widows, and orphans. There was also participatory and democratic management of the CBHI scheme, which increased trust and a sense of ownership by the entire community.^57^ However, a more recent analysis from a 2005–2006 nationally representative survey in Rwanda showed a positive relationship between socioeconomic status and enrollment in health insurance. While 50% of those in the richest quintile were insured, only 29% of those in the poorest quintile had insurance (*P* < .001).^58^ It is important to state that confounding factors that could have influenced the demand for health insurance, such as educational status, were not controlled for in the studies.

Our review also showed that as premium decreased the number of poor people who enrolled in a CBHI scheme increased. This shows that enrollment is price-elastic for the poor. Some households that could not afford the CBHI's initial high premium were able to enroll when the premium was lowered. Studies in Burkina Faso showed that providing subsidies increased enrollment for the poor^59^^–^^61^ while another study in China showed that at a lower premium, more households were willing to enroll in health insurance.^32^ Additionally, a study in Burkina Faso showed that with the onset of subsidies for the poor in the Naouna district in 2007, the proportion of the poor who were enrolled in the CBHI scheme increased from 1.1% in 2006 to 11.1% in 2007.^61^ Furthermore, in 2006 only 3.4% to 4.9% of all the insured were from poor households, but this increased to 26.0% to 28.8% in 2007.^60^^,^^61^ Another study in Burkina Faso showed that the price elasticity of the demand for CBHI was close to 1.^59^ Similarly, in China a study in 2002 showed that with a premium of 10 Yuan per year 76% of people were willing to join the CBHI. However, with a premium of 20 Yuan only 43% were willing to join the scheme.^32^

As premium decreased, the number of poor people who enrolled in community-based health insurance increased.

#### Enrollment in CBHI and Co-Pays

Studies in China showed that co-pays may be a disincentive for poor households to join CBHI schemes. An analysis of 4-year panel data on a voluntary CBHI scheme in rural China (Rural Mutual Health Care) showed that the low-income group was less likely to join the subsidized CBHI scheme than the middle- and high-income groups. One of the reasons that was given for this was that the co-pays might be too high for some poor people who then choose not to join.^74^ Another study that looked at the impact of China's cooperative medical scheme in rural communities showed that although premiums are highly subsidized for the poor, the scheme did not lead to improved use of services among the poorest 10% of the population. This was attributed to the co-pays at the point of accessing the services.^75^

Co-pays may be a disincentive for poor households to join community-based health insurance schemes.

To summarize, in the absence of factors that might negatively influence enrollment into CBHI, the rich are more likely to enroll into CBHI than the poor. In addition, CBHI enrollment is price-elastic, and the higher the premium, the smaller the number of people that will enroll in the scheme. Furthermore, we also saw that in Rwanda, supporting the poor to pay premiums removes the relationship between socioeconomic status and actual enrollment in CBHI. Finally, co-pays could negatively affect enrolment in CBHI and use of services even after enrollment.

### Socioeconomic Status and Use of Health Care for Enrollees

There were mixed results on the effect of socioeconomic status on use of health care among those enrolled in CBHI (Table 3). Studies in Burkina Faso showed that rich enrollees used health care more than poor enrollees.^51^^,^^62^ In Burkina Faso, the poor who were enrolled in the CBHI scheme had a 50% lower odds of using health services compared with the rich who were enrolled in the scheme.^51^ In another study in Burkina Faso, outpatient visits were 40 percentage points higher among the insured than the uninsured. However, this difference was only significant among the richest wealth quartile. This showed that the insurance scheme generally benefitted the rich more. Although there were no co-pays in the CBHI scheme in Burkina Faso and the insurance scheme covered essential drugs and referrals to the district hospital, the non-significant utilization by the poor might be due to other non-financial barriers to utilization such as distance from health facilities.^62^

**TABLE 3.** Summary of Studies on Community-Based Health Insurance Utilization or Drop-Out

Study	Country	Date of Data Collection	Sample Size^a^	Urban/ Rural	Study Design	Utilization or Drop-Out
Franco LM et al. (2008)^56^	Mali	2004	2,280	Both	Post with control	Insured were more likely to utilize health services
Schneider P et al. (2004)^57^	Rwanda	2000	2,518	Rural	Post with control	Utilization of health services by enrollees not associated with socioeconomic status
Gnawali DP et al. (2009)^62^	Burkina Faso	2006	990	Both	Post with control	Outpatient visits in insured 40% higher than in uninsured
Chankova S et al. (2008)^63^	Ghana, Mali, Senegal	Not stated	5,545	Both	Post with control	No difference in utilization based on socioeconomic status in the insured
Kent Ranson M et al. (2006)^64^	India	2003	3,844	Both	Post with control	Submission of claims for reimbursement was inequitable in rural areas; the rich were significantly more likely to submit claims than the poorest
Kent Ranson M (2004)^65^	India	2000	700	Both	Post with control	No significant difference in hospitalization among the different wealth quintiles
Dong H et al. (2009)^66^	Burkina Faso	2006	1,309	Both	Post with control	No statistically significant difference in the drop-out rate between income groups
Mladovsky P (2014)^67^	Senegal	2009	382	Both	Post with control	Those who dropped out were poorer than those who did not although this was not statistically significant

a Sample size is the number of households.

A study in Rwanda did not find any difference in health service utilization between rich and poor enrollees.^57^ In addition, household wealth quintiles in Mali did not show any consistent pattern of association with use of health services among those enrolled in the mutual health organization.^56^ Furthermore, studies in Burkina Faso and Rwanda showed that for the most part enrollment in CBHI schemes led to increased utilization of health services among the enrolled compared with the unenrolled.^51^^,^^57^^,^^62^ However, a study in Senegal did not find any difference in utilization between the insured and uninsured.^63^ This was attributed to the 25% to 50% co-payment for outpatient care in the Senegal CBHI scheme.

#### Utilization of Health Care and Reimbursement After Paying Out of Pocket

CBHI schemes that reimburse enrollees after paying for services out of pocket seem not to favor the poor. A study of the Self-Employed Women's Association (SEWA) insurance scheme in India, a CBHI scheme whereby members settle their hospital bills out of pocket and are reimbursed by the insurance scheme, suggested that there was an inequitable submission of claims among rural members. The mean socioeconomic status of rural claimants was significantly higher than the mean socioeconomic status of all rural members of the scheme. The poorest 30% of the members accounted for only 20% of the claims. Qualitative data revealed that the poorest in the rural communities might lack the money to pay bills at the time of hospitalization and so will use less of the services. Furthermore, the poor may also not be literate enough to fill the insurance claim form and so do not apply for reimbursement even after being hospitalized.^64^

However, in another SEWA survey there was no significant difference in hospitalization among the different wealth quintiles. This could be due to the small sample; there were only 28 admissions among the SEWA respondents in the 1-year recall period.^65^ In addition, there was no difference in hospitalization between the insured and uninsured.^65^ This could also be explained by the fact that the insured still need to pay out of pocket and be reimbursed later.

In summary, being insured was found to increase use of services compared with being uninsured. Although there were inconsistent findings on the relationship between socioeconomic status and use of health services among those enrolled in CBHI schemes, some studies showed lower use of services among the poor than the rich, which could be due to co-payments, travel costs, or non-financial barriers to use of services. Additionally, schemes that reimburse enrollees after they pay for services out of pocket seem to decrease use of services by the poor.

Some studies showed lower use of services among poor enrollees than rich enrollees, which could be due to co-payments, travel costs, or non-financial barriers to use of services.

### Socioeconomic Status and Drop-Out From CBHI

We noticed a high drop-out rate from CBHI schemes in the studies included in our study (Table 3). Although in the Nouna (Burkina Faso) CBHI scheme there was no statistically significant difference in the drop-out rate between income groups, the main reason people gave for dropping out was lack of money to pay the premium (28%) followed by dislike of medical staff behavior (19%).^66^ The drop-out rate from the Nouna district CBHI scheme in Burkina Faso was 31% in 2005 and 46% in 2006 for all the enrollees.^66^

Similarly, in a survey of 382 households in Senegal, the overall drop-out rate from the CBHI scheme was 72%. Those who dropped out were poorer than those who did not, although the difference was not statistically significant. The lack of statistical significance could be due to the small sample size.^67^

In summary, there is a high drop-out rate from CBHI schemes mainly due to inability or unwillingness to continue paying premiums. This affects all income groups and calls into question the effectiveness of CBHI programs as a means of achieving universal health coverage in low- and middle-income countries.

There is a high drop-out rate from community-based health insurance schemes mainly due to inability or unwillingness to continue paying premiums.

### POLICY IMPLICATIONS

As we stated earlier, a general taxation scheme is more desirable for providing comprehensive health insurance coverage for families, but this is not feasible in several low- and middle-income countries because people are poor and many work in the informal sector, which makes revenue collection difficult.^6^ This has led some poor countries to choose community health insurance schemes as an alternative way of providing access to basic health care for those in rural communities and the informal sector.^7^^,^^8^

The idea of CBHI schemes in most low- and middle-income countries is to provide improved health care access by the poor who might not be able to purchase private insurance or pay out of pocket for services. That explains why many of the CBHI schemes are located in areas where people are poor or work in the informal sector of the economy. However, for the scheme to be effective in achieving its goal of reaching the poor, several program features must be carefully designed. From our review, the measures in designing a CBHI scheme that would be beneficial to the poor include:

1. Offering flexible payment plans
2. Providing premium subsidies for the poor
3. Eliminating co-pays for the poor
4. Removing or reducing the waiting period after premium payment
5. Avoiding making patients pay out of pocket for services and getting reimbursed later

#### Flexible Payment Plans

A flexible payment plan or schedule in which the poor can pay in installments would be beneficial to the poor, although this might be administratively more expensive for programs to implement. Giving people the option to pay monthly, quarterly, or semiannually has been shown to help the poor pay their premium.^57^^,^^76^ This is because some of the poor who might be interested in enrolling into the scheme do not have the money to pay the yearly premium at one time. Under this flexible payment plan, families would be allowed to pay in installments and would be covered by the scheme once they complete their payment. This has been used in Rwanda and has been partly associated with the success of the scheme in covering the poor in the country.^57^ Families who are covered by the scheme can also start paying in installments for the next year. This would help reduce the high drop-out rate currently seen in CBHI schemes, which occurs because families do not have money to pay their yearly premiums when due. This might also be useful in rural communities where peasant farmers can start paying in installments for the next year once they sell their crops during the harvest season.

Giving people the option to pay monthly, quarterly, or semiannually has been shown to help the poor pay their health insurance premium.

#### Premium Subsidy for the Poor

Enrollment into a CBHI scheme is highly price-elastic for the poor, meaning that with highly subsidized premiums, more poor people will enroll in CBHI. The big questions are how to fund the subsidy and how to identify the poor. For nationally supported community health insurance schemes, government might subsidize the poor using money raised from taxes and external donors. A challenge is that not all countries might mobilize the financial resources to subsidize premiums for the poor in the face of other competing priorities. However, countries should prioritize premium subsidies if they want to increase scheme participation by the poor.

For small local CBHI schemes where funds are pooled at the community level, it becomes more difficult to raise money to subsidize the poor. Since studies have shown that the rich are willing to cross-subsidize the poor, one way is to have a progressive premium where the rich pay a little extra to cover the premium for very poor families. This is unlike many current CBHI schemes in which everyone pays a flat premium rate irrespective of income. Organizations such as churches, clubs, wealthy individuals in the community, and international donors can also be approached to make donations to subsidize the poor or to take up the premiums of specific very poor families who are willing to be so supported. This proved to be effective in the early stages of the CBHI scheme in Rwanda.^57^

The bigger question, however, is how to identify the poor who will receive the subsidy. Four common ways of identifying the poor include (1) means testing (identifying the poor using self-reported income or expenditure), (2) proxy means testing (classifying socioeconomic status based on ownership of assets and access to services), (3) geographical targeting (classifying people based on where they live, e.g., urban slums as poor), and (4) community wealth ranking (community members identify poor households based on their own definitions and perceptions)^77^^–^^81^ (Table 4).

**TABLE 4.** Methods of Identifying the Poor

Method	Ideal Condition to Use	Drawbacks
Means testing	When cost is not a consideration	Very expensive
Proximal means testing	Low-poverty incidence in urban areas	Expensive, measures relative poverty
Geographic targeting	High-poverty incidence in both urban and rural areas	Could lead to the non-poor who live in poor neighborhoods being exempted from premium
Community wealth ranking	Low-poverty incidence in rural communities	Measures relative poverty, cannot be used where community ties are weak

Adapted from Umeh CA (2017).^81^

In means testing, a questionnaire, such as the Living Standards Measurement Survey (LSMS) developed by the World Bank, is used to collect detailed information on household expenditure and consumption. Means testing is expensive because it involves collecting very detailed data through a household survey. This is in addition to other challenges such as the difficulty of assigning monetary value to food that local farmers harvest from their farms and recall bias for expenditures.^78^^–^^80^

Proximal means testing is being increasingly used to measure household socioeconomic status. Data on ownership of assets and access to services are collected from households, which are then used as proxies to determine household socioeconomic status. Some drawbacks of proximal means testing include cost of survey, inconclusive evidence that assets are good proxies of socioeconomic status, and the possibility of wrongly classifying the poor as not poor.^78^^–^^80^

In geographical targeting, families are classified as rich or poor based on the neighborhood in which they live. For example, families living in urban slums would be classified as poor. It could also involve the use of national survey data such as Demographic and Health Survey data to identify poor communities. However, geographical targeting could lead to the poor who are living in non-poor neighborhoods being excluded from the subsidy while the non-poor living in poor neighborhoods receive the subsidy that they do not need.^78^^–^^80^

In community wealth ranking, the community decides on the criteria that will be used to categorize the poor in the community. Then the community chooses key informants who have lived in the community for a long time and know all the households. The key informants individually categorize the families into different wealth groups. Then all the key informants meet to reach a consensus on the wealth categories of all the families. The advantage of this is that it is a fast and cost-effective way of assessing poverty level in the community. In addition, it is done by the community and because of the community participation, it will be easy for community members to agree to cross-subsidize the poor.^59^^–^^61^ The challenge with the community wealth ranking approach is that it might be difficult to use in urban areas where community ties are weak and people might not know each other very well.^61^ Another challenge with community wealth ranking is that it measures relative poverty. So someone who might be seen as poor in one community might not be identified as poor in another community depending on the average level of wealth in the different communities.

One particular method of assessing the poor might not work in all settings. To improve the ability to correctly identify the poor in a cost-effective manner, countries can combine more than one means of identifying the poor, such as first identifying the poor using community wealth ranking or geographical targeting and then using means testing or proximal means testing to screen those identified. It is left to the CBHI schemes to discover the method(s) that will best work for them in the specific locations and circumstances where they operate.

To improve the ability to correctly identify the poor cost-effectively, countries can combine more than one means of identifying the poor.

#### Removal of Co-Pays for the Poor

Although co-pays are put in place to prevent excessive use of health care services by those who do not need them (moral hazard), it is detrimental to the poor who really need the health care services but cannot afford the co-pay.^74^^,^^75^ Some might argue that removal of co-pay for the poor will lead to moral hazard for the poor, but that argument pales in light of the fact that there are other non-financial barriers that stop the poor from overusing health care services. Such things as transportation cost and opportunity cost of the time spent in the hospital are already barriers to excessive use of health care services by the poor.^82^

#### Removal or Reduction of Waiting Period After Premium Payment

In a bid to reduce adverse selection, some CBHI schemes introduce a waiting period (usually 3 months) between the payment of premium and coverage by the insurance scheme.^62^ However, the waiting period seems to adversely affect the poor more than the rich. This is because after paying for the premium, the poor might not have money to pay out of pocket during the waiting period. Although waiting periods are being used by many health insurance schemes to prevent adverse selection, we could not find studies that evaluated their effectiveness in preventing adverse selection. However, it was shown not to be effective in Burkina Faso^83^ as there was still adverse selection even with the waiting period in place.

CBHI schemes should consider the use of other methods that have been shown to be effective and are less harmful to the poor in preventing adverse selection. The use of enrollment at the household level where everyone in the household must be enrolled has been shown to be effective in reducing adverse selection.^84^ However, this did not eliminate adverse selection in some studies, because some households did not truly enroll all their household members.^83^^,^^85^ Another way to deal with adverse selection is to make signing up for insurance compulsory for everyone, with premium subsidies for the poor.^86^

#### Reimbursement of Expenses After Use of Services

The SEWA insurance scheme in India that reimbursed expenses after payment seemed not to favor the poor as there was an inequitable submission of claims, with the poorest 30% submitting fewer claims than the richer members.^64^ Although direct payment may potentially give consumers some leverage over provider quality, CBHI schemes that are structured in that way might be detrimental to the poor. One way to deal with this is for the insurance schemes to have an agreement with a network of health facilities and have their members receive treatment from those health facilities. Instead of the members paying out of pocket and being reimbursed later, the health facilities bill the insurance scheme directly and receive payment for the service from the insurance scheme. Insurers might also use the capitation payment method, which has been used in some CBHI schemes.^76^

## CONCLUSION

Achieving universal health coverage in low- and middle-income countries through CBHI schemes will be a difficult feat. For CBHI schemes to succeed in providing access to health care by the poor, and especially the poorest of the poor, such programs need to provide a subsidized premium for the poor and not to charge a premium at all for the poorest of the poor. In addition, providing flexible premium payment plans will help improve enrollment into CBHI schemes. Furthermore, removal of co-pays (especially) for the poor and removal of the waiting period between payment of premium and coverage by the health insurance scheme are steps that will be very beneficial to the poor. It is important to state that our recommendations are subject to the context of different countries in which the CBHI schemes operate.

## Notes

**Peer Reviewed**
